# Perceptions of tap water associated with low-income Michigan mothers’ and young children’s beverage intake

**DOI:** 10.1017/S1368980022001136

**Published:** 2022-10

**Authors:** Katherine W Bauer, Heidi M Weeks, Michelle Clayson, Belinda Needham

**Affiliations:** 1 Department of Nutritional Sciences, University of Michigan School of Public Health, 1415 Washington Heights, Ann Arbor, MI 48104, USA; 2 Department of Epidemiology, University of Michigan School of Public Health, Ann Arbor, MI, USA

**Keywords:** Tap water, Beverage intake, Infant feeding

## Abstract

**Objective:**

To quantify perceptions of tap water among low-income mothers with young children residing in Michigan and examine associations between perceptions of tap water, mothers’ and young children’s beverage intake, and mothers’ infant feeding practices.

**Design:**

Cross-sectional study.

**Setting:**

Online survey.

**Participants:**

Medicaid-insured individuals who had given birth at a large Midwestern US hospital between fall 2016 and fall 2020 were invited by email to complete a survey in winter 2020 (*N* 3881); 15·6 % (*N* 606) completed eligibility screening, 550 (90·8 %) were eligible to participate, and 500 (90·9 %) provided valid survey data regarding perceptions of tap water, self and child beverage intake, and infant feeding practices.

**Results:**

Two-thirds (66·2 %) of mothers reported that their home tap water was safe to drink without a filter, while 21·6 % were unsure about the safety of their home tap water. Mothers’ perceptions of their home tap water were associated with their own tap and bottled water intake and their young children’s tap water and bottled water intake. Mothers with more negative perceptions of tap water in general, independent of their perceptions about their home tap water, consumed more bottled water and sugar-sweetened beverages, and their young children drank bottled water and fruit drinks more frequently. Few associations were observed between mothers’ perceptions of tap water and infant feeding practices.

**Conclusions:**

Uncertainty about tap water safety and negative perceptions of tap water are common among low-income Michigan mothers. These beliefs may contribute to less healthful and more costly beverage intake among mothers and their young children.

Access to safe, reliable and affordable water is essential for human health. Humans require water for nearly all bodily processes including regulation of blood pressure, body temperature, metabolism, and cognitive function, and tap water is recommended as the primary beverage to meet daily water needs^(1)^. In nearly all communities in the USA, tap water meets the Environmental Protection Agency’s standards and is available at low cost^(2)^. Despite this near-universal safe access to tap water, over three-fourths of children do not meet the Institute of Medicine’s dietary reference intake recommendation for total water intake^(3)^ and nearly 50 % of water intake among US adults is from bottled water^(4)^.

In a recent nationally representative survey, 11 % of US parents were unsure whether their water was safe to drink^(5)^. Trusting that one’s water supply is safe is fundamental to relying on tap water for consumption^(2)^ and perceptions of tap water relate closely with intake of tap water. For example, adults who perceive that tap water is not safe to drink consume less tap water^(6)^ and children and adolescents whose parents believe bottled water is safer than tap water are more likely to drink bottled water^(7)^. Consuming bottled water is generally less favourable than consuming tap water in terms of health, cost and environmental impacts^(2)^. Individuals with high distrust of tap water may also substitute non-water beverages for tap water; however, evidence for this phenomenon is limited. Specifically, two studies have identified that distrust of tap water is associated with greater intake of sugar-sweetened beverages (SSB) among Hispanic/Latinx children and adults^(8,9)^. This is concerning as excess sugar intake, particularly via SSB, contributes to dental caries, obesity, diabetes and other chronic diseases^(10)^.

Communities in Michigan, unfortunately, are the ideal populations among which to examine the dietary impacts of diverse perceptions of tap water. In 2014, Flint Michigan’s state-appointed city manager switched the source of drinking water from the Detroit water system to the Flint River to save money. This decision resulted in lead contamination of the city’s drinking water. The effects of the Flint Water Crisis have rippled through Michigan, and since 2016, several other water systems across the state have been identified as lead-contaminated^(11)^. Adding insult to injury, in 2018 many of the state’s water systems were identified as being contaminated with polyfluoroalkyl substances, which contribute to a host of health problems^(12)^. In total, media reports suggest that distrust of tap water and distrust of government officials to keep tap water safe are high among Michigan residents^(13)^. Nationally, the percentage of adults and children drinking tap water has decreased dramatically since the Flint Water Crisis, particularly among individuals of colour^(14)^. Further, while avoidance of tap water had been decreasing among US children prior to the Flint Water Crisis, more children, specifically Black and Hispanic children, report avoiding tap water after the Flint Water Crisis^(15)^. While these trends are informative with respect to the potential effects of the Flint Water Crisis on water consumption nationwide, these studies were not able to link individuals’ perceptions of water with their dietary behaviours

The objective of the current study is therefore to quantify perceptions of home tap water and tap water in general among low-income Michigan mothers and examine associations between tap water perceptions and beverage intake among mothers and their young children (aged 0 through 4 years). Based on previous literature^(7–9)^, we hypothesised that mothers who felt their tap water was unsafe and those with negative perceptions of tap water would consume bottled water more frequently than tap water, their children would consume bottled water more frequently than tap water (including using bottled water used to mix formula), and both mothers and children would consume SSB more frequently. None of the previous studies linking perceptions of tap water to beverage intake have focused on children under 5 years of age and very little is known about how mothers’ perceptions of tap water are associated with breast-feeding or using tap water to mix formula, behaviours that have significant economic implications for low-income families. Study findings will add to our understanding of how perceptions of tap water are associated with beverage consumption, as well as expand our perspective on the potentially diffuse dietary impacts of tap water distrust. The knowledge gained from this study will inform culturally tailored policy, systems, and environmental interventions to improve tap water safety, reduce concerns about tap water, and promote healthier maternal nutrition and child feeding practices.

## Materials and methods

### Study design and participant recruitment

Data for the current analysis were obtained from a survey of low-income mothers living in Michigan but outside of Flint, the primary aim of which was to understand the indirect effects of the Flint Water Crisis on Michigan residents. Data were collected in November and December 2020 via a Qualtrics-based online survey. Participant recruitment was conducted in partnership with the university’s Data Office for Clinical and Translational Research (DOCTR), which supports the enrolment of health system patients in clinical research. To recruit low-income mothers, DOCTR identified individuals who had given birth at the university’s hospital since 1 September 2016, were insured by Medicaid (requiring a household income up to 200 % of the federal poverty line) and had an email address in their medical record. Patients identified through this query were sent an email inviting them to complete the study’s eligibility screener via a personalised, one-time-use link. The study was advertised as seeking to understand women’s beverage choices.

Eligibility criteria included participants who identified as female, were 18–45 years old at the time of screening, lived in the state of Michigan but not Genesee County (the location of Flint, Michigan) and were able to complete the survey in English. Individuals who did not meet these criteria or who skipped any of the screening questions were ineligible. Qualtrics’ Captcha feature was used to minimise the risk of false participants. Eligible individuals were automatically progressed to the study survey and those who completed at least 78 % of the study survey received a $20 gift card as compensation. The study was deemed exempt by the university’s Institutional Review Board.

Study invitations were emailed to 3881 individuals, 606 completed the eligibility screener and 550 were eligible to participate. Among those 550 individuals, 26 either did not complete the survey or did not provide any data on perceptions of tap water, 21 were identified as providing invalid survey responses and 3 reported that they did not have any children. Excluding these individuals led to a final analytic sample of 500 participants. Because study recruitment was tied to having given birth at the university’s hospital, most participants lived in southeastern Michigan.

### Assessments and measures

Selection of survey constructs and questions was informed by existing literature, and a focus group conducted in October 2019 with leaders of Michigan-based organisations that serve women and children during which low-income families’ experiences with tap water safety were discussed. When possible, measures were used or adapted from existing measures. Survey questions were then reviewed for comprehension and applicability to diverse populations by survey development experts at the University of Michigan’s Institute for Survey Research.

### Perceptions of tap water

#### Safety of home tap water

To determine whether mothers’ home tap water was safe to drink, participants were asked the question, ‘Is the tap water in your home safe to drink?’ and provided response options, ‘Yes’, ‘Only with a filter’, ‘No’ and ‘Not sure’. This question was used by the C.S. Mott Children’s Hospital’s National Poll on Children’s Health^(5)^.

#### Perceptions of home tap water

Mothers’ broader perceptions of the tap water in their home were assessed by responses to five statements adapted from Doria et al.^(16)^ assessing happiness with their home tap water’s taste, colour, and odour, and belief about their home tap water being contaminated. Response options ranged from ‘strongly agree’ to ‘strongly disagree’ on a five-item Likert scale. Items were re-coded so that higher scores indicated more negative perceptions of home tap water, and a mean summary score across the five items was calculated.

#### Perceptions of tap water in general

Mothers’ perceptions of tap water in general were assessed by examining agreement with five statements adapted from Doria et al.^(16)^ including that people they know and the media make negative comments about tap water, tap water has caused health problems for themselves or a family member, and they desire to drink bottled water more often than they currently do. Responses on a five-item Likert scale ranging from ‘strongly agree’ to ‘strongly disagree’ were re-coded such that higher scores indicated more negative general perceptions. A mean summary score across the five statements was then calculated.

#### Mothers’ beverage intake

Mothers were asked three questions about their water intake when at home: ‘How often do you drink unfiltered tap water at home?’, ‘How often do you drink filtered tap water at home?’ and ‘How often do you drink bottled water at home? Do not include carbonated water like sparkling water, seltzer or club soda’. These questions were adapted from Hobson et al.^(17)^, and response options included ‘always’, ‘sometimes’, ‘rarely’ and ‘never’. To assess SSB intake, mothers were asked three questions regarding their past month frequency of intake of regular soda, sweetened fruit drinks and other sugar-sweetened drinks. Six response options ranged from ‘Never or less than 1 time per week’ to ‘2+ times per day’. These questions were adapted from the national Behavioral Risk Factor Surveillance Survey^(18)^. Responses were coded to represent number of times per week and summed across the three questions to calculate total weekly SSB intake.

#### Children’s beverage intake

Mothers who reported having a child aged 1 through 4 years were asked to report their child’s past month frequency of intake of beverages including sweetened and unsweetened milk, 100 % fruit juice, fruit drinks, regular soda, and tap, filtered, and bottled water. Beverages queried about were informed by Marshall et al.^(19)^ and the Beverage Questionnaire for Preschoolers (BEVQ-PS)^(20)^. Six response options ranged from ‘Never or less than 1 time per week’ to ‘2+ times per day’ as used to measure intake frequency in the BEVQ-PS, and responses to each item were re-coded to represent number of times per week. Mothers with multiple children between 1 and 4 years old were asked to report on their oldest 1 through 4-year-old child’s intake.

#### Infant feeding practices

Mothers who reported having a child less than 1 year of age were asked a series of questions about their feeding practices. If mothers had more than one child in this age range, they were asked to report on their oldest infant. First, mothers were asked, ‘Other than solid foods (such as purees, puffs or Cheerios), how are you currently feeding your infant?’ with response options including, ‘Breast-feeding only’, ‘Formula feeding only’, ‘Both breast and formula feeding’, and ‘Other (please describe below)’^(21)^. Responses were collapsed to capture exclusive breast-feeding *v*. all other types of feeding. If mothers reported any formula feeding, they were then asked whether they use powdered formula, liquid/ready-to-feed formula or a combination of both. If they then reported any powdered formula use, they were asked which type of water they use most often to make powdered formula with response options of ‘Water straight from the tap’, ‘Filtered tap water’, ‘Boiled tap water’, ‘Regular bottled water’, ‘Bottled water for babies (with fluoride)’ and ‘Other (please describe below)’. ‘Other’ responses were re-coded by study investigators into one of the first five response options. Response options 1–3 and 4 and 5 were then combined to capture tap *v*. bottled water. Finally, mothers who reported any breast-feeding were asked, ‘How much did the safety of your tap water influence your decision to breastfeed?’ with four response options ranging from ‘a great deal’ to ‘not at all’. Responses were collapsed to capture any influence of tap water safety on breast-feeding decisions *v*. no influence.

#### Sociodemographic and home water characteristics

Mothers reported the ages of their children under 5 years in months and years, whether each child was covered by Medicaid, their race/ethnicity, their highest level of educational attainment, and whether they or the children in their household participate in food assistance programmes including Special Supplemental Nutrition Program for Women, Infants, and Children (WIC) and the Supplemental Nutrition Assistance Program (SNAP). Mothers also completed a two-item measure to assess risk of household food insecurity^(22,23)^. Finally, mothers were asked the source of tap water in their home with options, including ‘City water system’, ‘Well water’, ‘Rural water system’, ‘Other’ and ‘Not sure’^(5)^, as well as an open-ended question about how many dollars they spend on bottled water for their home each month.

### Statistical analysis

Univariate statistics were calculated for all sociodemographic characteristics among all mothers as well as mothers of infants and mothers of 1 through 4-year-olds separately as subsequent analyses focused on these two age groups of children. Differences in all mothers’ perceptions of tap water were examined across sociodemographic characteristics using chi-square or ANOVA, as appropriate. Because mothers’ spending on bottled water was not normally distributed, associations with perceptions of tap water were tested using Kruskal–Wallis tests or Spearman’s correlations. Regression models were then built to examine associations between perceptions of tap water, mothers’ beverage intake, children’s beverage intake and infant feeding practices. Generalised linear models were used for continuous outcomes (mothers’ tap, filtered and bottled water intake), negative binomial regression for count outcomes (mothers’ SSB intake and children’s beverage intake) and logistic regression for binary outcomes (infant feeding practices). All models were adjusted for mothers’ race/ethnicity, age, educational attainment, food security status and water source. Given the number of tests, *P*-values < 0·01 were used to indicate statistical significance to reduce Type 1 error. All analyses were conducted using SAS 9.4 (Cary, NC).

## Results

Among the 500 mothers in the study, 147 had at least 1 child less than 1-year-old and 417 had at least 1 child aged 1 through 4 years old (Table 1). Approximately half (56·2 %) of mothers identified as non-Hispanic White, 30·2 % as non-Hispanic Black, 10·6 % as Hispanic/Latina and 3·0 % as another race/ethnicity. Most mothers (55·4 %) had completed some college or had an associate degree and 43·1 % were identified as food-insecure. Eighty-five per cent of households had at least one child aged 0–4 years enrolled in Medicaid, 52·3 % of households participated in WIC and 43·7 % of households received SNAP benefits. Seventy-six per cent of mothers reported that the city supplies their home tap water with the next most frequent response being well water, which 15 % of mothers reported. Median spending on bottled water for the home was $20/month.

**Table 1:** Sociodemographic characteristics of mothers overall and by child age

	All mothers (*n* 500)		Mothers with infants (*n* 147)^*^		Mothers with 1–4-year-olds (*n* 417)^*^	
	%	%	%	%	%	%
Mothers’ age in years						
Mean	30·8		29·9		30·9	
sd	5·3		5·5		5·3	
Mothers’ race/ethnicity (%)						
Non-Hispanic White	56·2		56·5		56·1	
Non-Hispanic Black	30·2		32·7		30·5	
Hispanic/Latina	10·6		7·5		10·3	
Other race/ethnicity	3·0		3·4		3·1	
Mothers’ educational attainment (%)						
Did not finish HS/completed HS	20·8		25·9		20·6	
Some college/associate degree	55·4		50·3		55·4	
Bachelor’s degree	16·4		18·4		15·6	
Advanced degree	7·4		5·4		8·4	
Food-insecure (%)	43·1		44·2		42·0	
Child(ren) aged 0–4 years enrolled in Medicaid (%)	85·0		95·8		82·3	
Household member(s) participate in WIC (%)	52·3		70·1		50·2	
Household receives SNAP benefits (%)	43·7		51·0		42·3	
City supplies water (*v*. well, rural, other and not sure) (%)	76·0		80·1		75·2	
Monthly spending on bottled water, **$**						
Median	20·00		20·00		20·00	
IQR	8·00–30·00		10·00–30·00		6·00–34·00	

HS, high school; WIC, Special Supplemental Nutrition Program for Women, Infants, and Children; SNAP, Supplemental Nutrition Assistance Program; IQR, interquartile range.

* Not mutually exclusive, seventy-eight women had an infant and child between the age of 1 and 4 years.

Overall, 66·2 % of mothers reported that their home tap water was safe to drink without a filter (Table 2). Only a small number of mothers (1·8 %) reported their tap water was not safe to drink at all, whereas 10·4 % reported it was only safe with a filter. These categories were combined in further analyses. More than one in five mothers (21·6 %) were unsure whether their home tap water was safe to drink. No differences in mothers’ report of the safety of their home tap water nor their perceptions of their home tap water (e.g. its smell, taste and odour) were observed by race/ethnicity or educational attainment. In comparison, mothers’ perceptions of tap water in general differed by race/ethnicity and educational attainment with Black mothers and mothers without post-high school education reporting the greatest negative perceptions of tap water in general. Mothers identified as food-insecure were less likely than food-secure mothers to report that their tap water was safe to drink without a filter (57·2 % *v*. 72·9 %) and reported more negative perceptions of their home tap water and tap water in general. Mothers with well water and other non-city sources were also less likely to report their tap water was safe to drink without a filter (51·7 % *v*. 71·0 %) and mothers who reported that their tap water was not safe to drink or only safe with a filter spent $5 more per month on bottled water each month than other mothers. Further, although both perceptions of home tap water and perceptions of tap water in general were associated with mothers’ monthly spending on bottled water, general perceptions of tap water were more strongly correlated with spending on bottled water (*r* = 0·36, *P* < 0·0001) than perceptions of home tap water (*r* = 0·23, *P* < 0·0001).

**Table 2:** Differences in perceptions of tap water by select sociodemographic and personal factors among all mothers (*n* 500)

	Safety of home tap water							Negative perceptions of home tap water (range: 1–4)			Negative perceptions of tap water in general (range: 1–4)		
	Safe without filter (%)		No or only with filter (%)		Not sure (%)		*P*	Mean	sd	*P*	Mean	sd	*P*
Total	66·2		12·2		21·6		–	2·4	0·9	–	3·2	0·7	–
Mothers’ race/ethnicity							0·32			0·62			<0·0001
Non-Hispanic White	67·6		11·7		20·6			2·3	0·9		3·1	0·7	
Non-Hispanic Black	66·2		9·9		23·8			2·4	0·8		3·4	0·7	
Hispanic/Latina	58·5		17·0		24·5			2·3	0·8		3·2	0·9	
Other race/ethnicity	66·7		26·7		6·7			2·6	0·8		3·1	0·5	
Mothers’ education							0·26			0·39			0·004
Did not finish HS/completed HS	60·6		8·7		30·8			2·4	0·9		3·3	0·7	
Some college/associate degree	67·5		13·0		19·5			2·4	0·9		3·2	0·7	
Bachelor’s degree	68·3		14·6		17·1			2·2	0·9		3·0	0·7	
Advanced degree	67·6		10·8		21·6			2·4	1·0		3·0	0·8	
Household food security							0·001			0·0003			<0·0001
Food-secure	72·9		10·2		16·9			2·2	0·8		3·1	0·7	
Food-insecure	57·2		14·9		27·9			2·5	0·9		3·3	0·7	
Tap water source							<0·0001			0·09			0·005
City	71·0		9·0		20·1			2·3	0·8		3·2	0·7	
Well, rural, other and not sure	51·7		21·7		26·7			2·5	0·9		3·0	0·7	
	Median	IQR	Median	IQR	Median	IQR		Spearman’s r		*P*-value	Spearman’s r		*P*-value
Monthly spending on bottled water	$20·00	5·00–30·00	$25·00	10·00–50·00	$20·00	10·00–40·00	0·002	0·23		<0·0001	0·36		<0·0001

HS, high school; IQR, interquartile range.

After adjusting for mothers’ race/ethnicity, age, educational attainment, water source and food security status, mothers’ report of the safety of their home tap water was associated with the frequency with which they drank unfiltered water and bottled water (Table 3). For example, mothers who reported that their tap water was safe to drink without a filter drank bottled water less frequently than other mothers (mean (se) = 2·9 (0·1)), while bottled water intake was similar between mothers who reported their water was not safe or safe only with a filter (mean (se) = 3·4 (0·1)) and those who were unsure of the safety of their tap water (mean (se) = 3·3 (0·1)). Overall, mothers reported consuming SSB 6·3 (sd = 7·7) times/week and SSB intake did not vary by safety of home tap water.

**Table 3:** Associations between home tap water safety and mothers’ intake, children’s intake and infant feeding practices^†^

					Safety of home tap water					
					Safe without filter		No or only with filter		Not sure	
		*n* responses for outcome	Mean	sd	Adjusted mean	se	Adjusted mean	se	Adjusted mean	se
Mothers’ intake outcomes	Drinks unfiltered tap water (range: 1–4)	495	2·3	1·1	2·5	0·1^a^	1·5	0·1^b^	1·8	0·1^b^
	Drinks filtered tap water (range: 1–4)	494	2·4	1·2	2·5	0·1^a^	2·7	0·2^a^	2·2	0·1^a^
	Drinks bottled water (range: 1–4)	494	3·1	1·0	2·9	0·1^a^	3·4	0·1^b^	3·3	0·1^b^
			Mean	sd	IRR	99 % CI	IRR	99 % CI	IRR	99 % CI
	SSB intake (times/week)	490	6·3	7·7	Ref		0·76	0·46, 1·25	1·04	0·71, 1·52
Children’s intake outcomes	Drinks unfiltered tap water (times/week)	399	3·1	5·0	Ref		0·08	0·03, 0·24^**^	0·57	0·29, 1·14
	Drinks filtered tap water (times/week)	396	4·5	5·8	Ref		1·10	0·51, 2·37	0·66	0·35, 1·24
	Drinks bottled water (times/week)	400	6·0	5·7	Ref		1·49	0·88, 2·53	1·64	1·09, 2·45^*^
	Unsweetened Milk (times/week)	400	7·7	5·7	Ref		0·89	0·62, 1·27	0·97	0·73, 1·29
	Sweetened Milk (times/week)	396	2·0	3·5	Ref		0·87	0·38, 1·99	1·12	0·59, 2·15
	100 % juice (times/week)	399	3·8	4·0	Ref		0·92	0·58, 1·45	1·13	0·79, 1·62
	Fruit drinks (times/week)	400	1·4	2·5	Ref		0·66	0·29, 1·48	1·46	0·79, 2·68
	Soda/pop (times/week)	399	0·2	0·7	Ref		0·77	0·13, 4·58	1·11	0·28, 4·36
			%		OR	99 % CI	OR	99 % CI	OR	99 % CI
Infant feeding practices outcomes	Exclusively breastfeeds (%)	143	28·7		Ref		0·54	0·09, 3·36	1·13	0·30, 4·19
	Mothers using formula use bottled water to mix formula (%)	95	64·2		Ref		14·02	0·60, 330·38	1·63	0·33, 8·06
	Among mothers breast-feeding, water safety influenced breast-feeding (%)	69	31·9		Ref		34·74	1·60, 756·70^*^	5·37	0·59, 49·03

IRR, incidence rate ratio; SSB, sugar-sweetened beverage.

^a,b,c^ Mean values within a row with unlike superscript letters were significantly different (*P* < 0·01).

* Statistically significant at *P* < 0·01.

** Statistically significant at *P* < 0·0001.

† Models adjusted for mothers’ race/ethnicity, age, educational attainment, food security status and water source. Generalised linear models were used to estimate adjusted means, negative binomial regression models were used to estimate IRR and logistic regression models were used to estimate OR.

Children whose mothers reported that their tap water was not safe to drink or not safe without a filter drank unfiltered tap water 92 % less frequently than children of mothers who reported their tap water was safe without a filter (incidence rate ratio (IRR) (99 % CI) = 0·08 (0·03, 0·24)), but no differences in filtered water intake or bottled water intake were seen between these groups. Meanwhile, compared to children of mothers who reported their tap water was safe without a filter, children of mothers who were unsure about the safety of their tap water drank bottled water 64 % more frequently (IRR (99 % CI) = 1·64 (1·09, 2·45)). Finally, breast-feeding mothers who reported that their home tap water was not safe to drink or only safe with a filter were more likely to report that water safety influenced their breast-feeding as compared to mothers reporting safe home tap water; however, the CI around this estimate was quite large (OR (99 % CI) = 34·74 (1·60, 756·70)).

Finally, there was a modest association between mothers’ perceptions of home tap water and tap water in general (r = 0·46), and in regression models that mutually adjusted for these predictors, negative perceptions of home tap water were associated with less frequent unfiltered tap water intake (B (se) = –0·43 (0·06), *P* < 0·0001), while negative perceptions of tap water in general were associated with more frequent bottled water (B (se) = 0·53 (0·06), *P* < 0·0001). Additionally, each unit increase in negative perceptions of tap water in general was associated with a 56 % increase in mothers’ weekly frequency of SSB consumption (IRR (99 % CI) = 1·56 (1·18, 2·06)) (Table 4). Similarly, mothers’ negative perceptions of tap water in general, but not perceptions of home tap water, were associated with a greater frequency of children’s bottled water consumption (IRR (99 % CI) = 1·73 (1·32, 2·28)) and more frequent fruit drink consumption (IRR (99 % CI) = 1·54 (1·00, 2·36)). Meanwhile, mothers with more negative perceptions of their home tap water reported that their children consumed fruit drinks less frequently (IRR (99 % CI) = 0·69 (0·49, 0·97)). Mothers’ perceptions of their home tap water and tap water in general were not associated with odds of engaging in the infant feeding practices examined.

**Table 4:** Associations between mothers’ perceptions of tap water and mother and child beverage intake and infant feeding^†^

		Negative perceptions of home tap water		Negative perceptions of tap water in general	
Motders’ intake outcomes		B	se	B	se
	Drinks unfiltered tap water (range: 1–4)	−0·43	0·06^**^	−0·07	0·08
	Drinks filtered tap water (range: 1–4)	0·01	0·07	−0·08	0·09
	Drinks bottled water (range: 1–4)	0·01	0·05	0·53	0·06^**^
		IRR	99 % CI	IRR	99 % CI
	SSB intake (times/week)	0·92	0·74, 1·14	1·56	1·18, 2·06^**^
Children’s intake outcomes	Drinks unfiltered tap water (times/week)	0·69	0·45, 1·06	0·85	0·51, 1·41
	Drinks filtered tap water (times/week)	1·01	0·73, 1·40	0·84	0·56, 1·25
	Drinks bottled water (times/week)	1·05	0·85, 1·29	1·73	1·32, 2·28^**^
	Unsweetened milk (times/week)	0·97	0·83, 1·13	1·00	0·83, 1·21
	Sweetened milk (times/week)	0·98	0·68, 1·42	1·10	0·70, 1·74
	100 % juice (times/week)	0·94	0·78, 1·14	1·15	0·89, 1·49
	Fruit drinks (times/week)	0·69	0·49, 0·97^*^	1·54	1·00, 2·36^*^
	Soda/pop (times/week)	1·24	0·56, 2·75	1·16	0·41, 3·27
		OR	99 % CI	OR	99 % CI
Infant feeding practices outcomes	Exclusively breastfeeds	1·45	0·70, 3·02	0·76	0·30, 1·93
	Uses bottled water to mix formula	1·50	0·54, 4·18	3·42	0·98, 11·90
	Water safety influenced breast-feeding	1·67	0·61, 4·54	2·18	0·56, 8·51

B, β-estimate; IRR, incidence rate ratio; SSB, sugar-sweetened beverage.

* Statistically significant at *P* < 0·01.

** Statistically significant at *P* < 0·0001.

† Models mutually adjusted for the two predictors’ perceptions of home tap water and perceptions of tap water in general in addition to mothers’ race/ethnicity, age, educational attainment, food security status and water source. Generalised linear models were used to estimate β-estimates, negative binomial regression models were used to estimate IRR and logistic regression models were used to estimate OR.

## Discussion

Only 66 % of low-income Michigan mothers participating in the current study felt that their tap water was safe to drink without a filter, while 21·6 % were unsure whether their water was safe to drink. Uncertainty about the safety of home tap water was most prevalent among mothers experiencing food insecurity and those with well water. Uncertainty about tap water safety was in turn associated with greater bottled water consumption among mothers and children, which creates an economic burden for families^(2)^. Further investigation of individuals who are not sure whether their tap water is safe to drink is essential to identify the drivers of this uncertainty and effective approaches to mitigate it. Contrary to our hypothesis, safety of home tap water and mothers’ perceptions of their home tap water were not associated with SSB intake among mothers or children. However, mothers with negative perceptions of tap water in general reported more frequent SSB consumption. As previous research has only identified relationships between trust in tap water and SSB intake among Hispanic/Latinx populations^(8)^, together, these results suggest there may be unique social norms that contribute to individuals consuming SSB when distrust of water is high.

Previous studies suggest that distrust of tap water stems from factors including repeated community failures to regulate the safety of water, limited understanding of water treatment and monitoring processes, personal or historical experience with unsafe water supplies, and greater belief that sensory qualities of water (e.g. odour and taste) indicate contamination^(6,24)^. For families whose home tap water is safe, reducing uncertainty about its safety may be a potent approach to reduce the expense of purchasing bottled water among already nutritionally vulnerable populations. In the USA, public water systems are required to inform residents annually about the quality of their water via consumer confidence reports. However, there reports are largely inaccessible to individuals with lower literacy^(25)^. Revising consumer confidence reports to ease comprehension, in concert with promotion of low-cost home lead test kits and complementary counselling on tap water, for example through existing maternal and infant home visiting programmes^(26)^, may be successful approaches to reduce uncertainty about tap water safety.

While mothers’ perceptions of their home tap water did not vary by race, ethnicity or educational attainment, differences were observed in mothers’ perceptions of tap water in general by these sociodemographic characteristics. National and community-specific studies have documented that Black, Hispanic/Latinx and low socio-economic status individuals are more likely than White and high socio-economic status individuals to report that tap water is not safe to drink^(8,9,17,27,28)^ and are less likely to consume tap water^(14,15)^. Further, Black and Hispanic/Latinx adults in the USA are twice as likely as White adults to consume bottled water^(29,30)^. The current study builds on this literature by demonstrating distinctions between mothers’ perceptions of their home tap water *v*. tap water in general; these constructs appear to be differently socially patterned and have distinct associations with beverage intake. Specifically, factors such as negative messaging about tap water and friends’ and family members’ experiences with tap water, not mothers’ perceptions of the tap water in their own home, were positively associated with bottled water use and mother and child SSB intake. These findings suggest that efforts to build trust in tap water and modify beverage purchasing and consumption practices may be more effective among low-income communities and Black and Hispanic/Latinx communities by targeting social norms and media messages regarding tap water safety and the negative implications of bottled water use instead of, or in addition to, focusing on individuals’ perceptions of their home tap water.

To our knowledge, the current study is the first to examine how perceptions of tap water relate to breast and/or formula feeding practices among US women. Over 30 % of breast-feeding mothers reported that the safety of tap water influenced their decision to breastfeed, and this was most common among mothers who felt that their home tap water was not safe to drink or only safe with a filter. Contrary to our hypothesis, mothers’ perceptions of tap water safety were not associated with use of bottled water *v*. tap water to mix formula. However, our sample of formula feeding mothers was relatively small. Further research with larger samples of parents of infants is essential to strengthen our understanding of how perceptions of tap water may impact infant feeding.

While this study fills an important gap in our understanding of low-income mothers’ perceptions of tap water and their potential impacts, it was not without limitations. Participants were recruited via email, and data were collected via internet-based surveys, in part due to restrictions on in-person research during the COVID-19 pandemic. While every effort was made to ensure data quality, the most important being the use of a known participant pool and implementation of multiple data security methods, the study methods may have influenced who enrolled in the study. Further, mothers were recruited based on being publicly insured during pregnancy and had to self-identify as speaking English to be eligible for the study, thus limiting the socio-economic and cultural diversity of the sample. Additionally, our sample of mothers of infants was relatively small. Further studies focused on perceptions of tap water among mothers of infants are needed to guide policies and messaging regarding using tap water to feed infants.

## Conclusions

Distrust of tap water is high among low-income Michigan mothers, and this distrust may have important implications for mothers’ decisions regarding how to nourish themselves and their young children. Distrust and uncertainty about tap water were greatest among families of lower socio-economic status and/or those experiencing food insecurity, as well as Black and Hispanic/Latina participants. These families are the most vulnerable to the excess costs and health risks that accompany distrust of tap water and should be prioritised with respect to ensuring access to safe, secure, low-cost drinking water. Programmes and policies targeting both increasing consumers’ understanding of their tap water and broader community perceptions of tap and bottled water may work synergistically to increase trust in tap water among low-income families.
